# Ectopic expression of DnaJ type-I protein homolog of *Vigna aconitifolia* (*VaDJI*) confers ABA insensitivity and multiple stress tolerance in transgenic tobacco plants

**DOI:** 10.3389/fpls.2023.1135552

**Published:** 2023-04-19

**Authors:** Ranjana Gautam, Rajesh Kumar Meena, Sakshi Rampuria, Pawan Shukla, P. B. Kirti

**Affiliations:** ^1^ Department of Plant Sciences, School of Life Sciences, University of Hyderabad, Hyderabad, Telangana, India; ^2^ Department of Life Sciences and Biotechnology, Chhatrapati Shahu Ji Maharaj University, Kanpur, Uttar Pradesh, India; ^3^ Seri-Biotech Research Laboratory, Central Silk Board, Bangalore, India

**Keywords:** drought stress, heat stress, DnaJ, photosynthetic efficiency, tobacco, gene expression, *VaDJI*

## Abstract

Reduced crop productivity results from altered plant physiological processes caused by dysfunctional proteins due to environmental stressors. In this study, a novel DnaJ Type-I encoding gene, *VaDJI* having a zinc finger motif in its C-terminal domain was found to be induced early upon treatment with heat stress (within 5 min) in a heat tolerant genotype of *Vigna aconitifolia* RMO-40. *VaDJI* is induced by multiple stresses. In tobacco, ectopic expression of *VaDJI* reduced ABA sensitivity during seed germination and the early stages of seedling growth of transgenic tobacco plants. Concomitantly, it also improved the ability of transgenic tobacco plants to withstand drought stress by modulating the photosynthetic efficiency, with the transgenic plants having higher F_v_/F_m_ ratios and reduced growth inhibition. Additionally, transgenic plants showed a reduced build-up of H_2_O_2_ and lower MDA levels and higher chlorophyll content during drought stress, which attenuated cell damage and reduced oxidative damage. An analysis using the qRT-PCR study demonstrated that *VaDJI* overexpression is associated with the expression of some ROS-detoxification-related genes and stress-marker genes that are often induced during drought stress responses. These findings suggest a hypothesis whereby *VaDJI* positively influences drought stress tolerance and ABA signalling in transgenic tobacco, and suggests that it is a potential gene for genetic improvement of drought and heat stress tolerance in crop plants.

## 1 Introduction

The detrimental environmental factors coming under abiotic stresses include temperature extremes, salinity, drought and nutrient deficiencies, which limit normal growth and development in plants thereby reducing their productivity (He et al., 2018; Ma et al., 2020). Among them, drought and heat stresses are considered the two most important abiotic stresses limiting productivity due to their complex nature (Fahad et al., 2017; Dos Santos et al., 2022). Therefore, understanding the plant response mechanism associated with these stresses is fundamentally needed to protect the plants from climate vulnerability and sustain their productivity. Given their vulnerability to toxicity produced by abiotic stresses in nature, plants heavily rely on the intrinsic signalling network of adaptive mechanisms that operate at molecular and physiological levels, which vary from species to species (Bohnert et al., 1995; Zhu, 2016). Some signalling mechanisms regulate the folding, degradation and trafficking of proteins to ensure that protein homeostasis is maintained in plants under stressful conditions.

Generally, environmental adversity in plants causes denaturation of proteins; thus, the maintenance of proteins in their respective conformation and the prevention of protein aggregation are imperative for plant cell survivability under stress conditions (Nakajima and Suzuki, 2013). Molecular chaperones are proteins that are ubiquitous in nature and are shown to be involved in various stress-related functions and different cell-related processes including cytoprotection and modulate stress-related regulatory networks both at transcriptional and post-transcriptional levels (Al-Whaibi, 2011; Jacob et al., 2017; Zhang et al., 2020; Ling et al., 2021). They are involved in the protein folding and unfolding as well as repair of damaged proteins and their reactivation which constitute the mainstay of stress-related metabolism in plants (Parsell and Lindquist, 1993; Sun et al., 2021). Heat shock proteins (Hsps) or molecular chaperones have been classified into different families according to their sequence homology and molecular weight, including small Hsp, Hsp40, Hsp60, Hsp70, Hsp90 and Hsp100 (Gupta et al., 2010; Fan et al., 2017; Zarouchlioti et al., 2018; Wang et al., 2019a; Wang et al., 2020; Li et al., 2021).

Hsp70 proteins are ubiquitous and versatile proteins that are associated with various activities related to protein function including their folding and refolding, degradation and several other related activities. The J-domain-containing Hsp40 proteins are the main co-chaperones of the Hsp70 proteins and are involved in modulating their functions (Walsh et al., 2004; Kampinga and Craig, 2010). The J-domain of the proteins is involved in the interaction with the Hsp70 proteins and hence, the J-domain containing proteins exhibit different expression patterns and, protein structure and sequence, which control the stress ameliorating activity of the Hsp complexes (Pulido and Leister, 2018). They are generally expressed as families of proteins and the domains other than the J-domain in them drive the “specificity of the system by delivering specific substrate polypeptides or by attracting Hsp70 partners to their site of actions” (Kampinga et al., 2019). These Hsp40 proteins are also expressed in mammalian and avian cell lines under heat-stress conditions (Ohtsuka et al., 1990). The downregulation of a DnaJA1 protein has been linked to pancreatic cancer in humans and its overexpression reduced the detrimental effects of the c-Jun protein and consequently enhanced cell survival (Stark et al., 2014).

**Figure 1:**
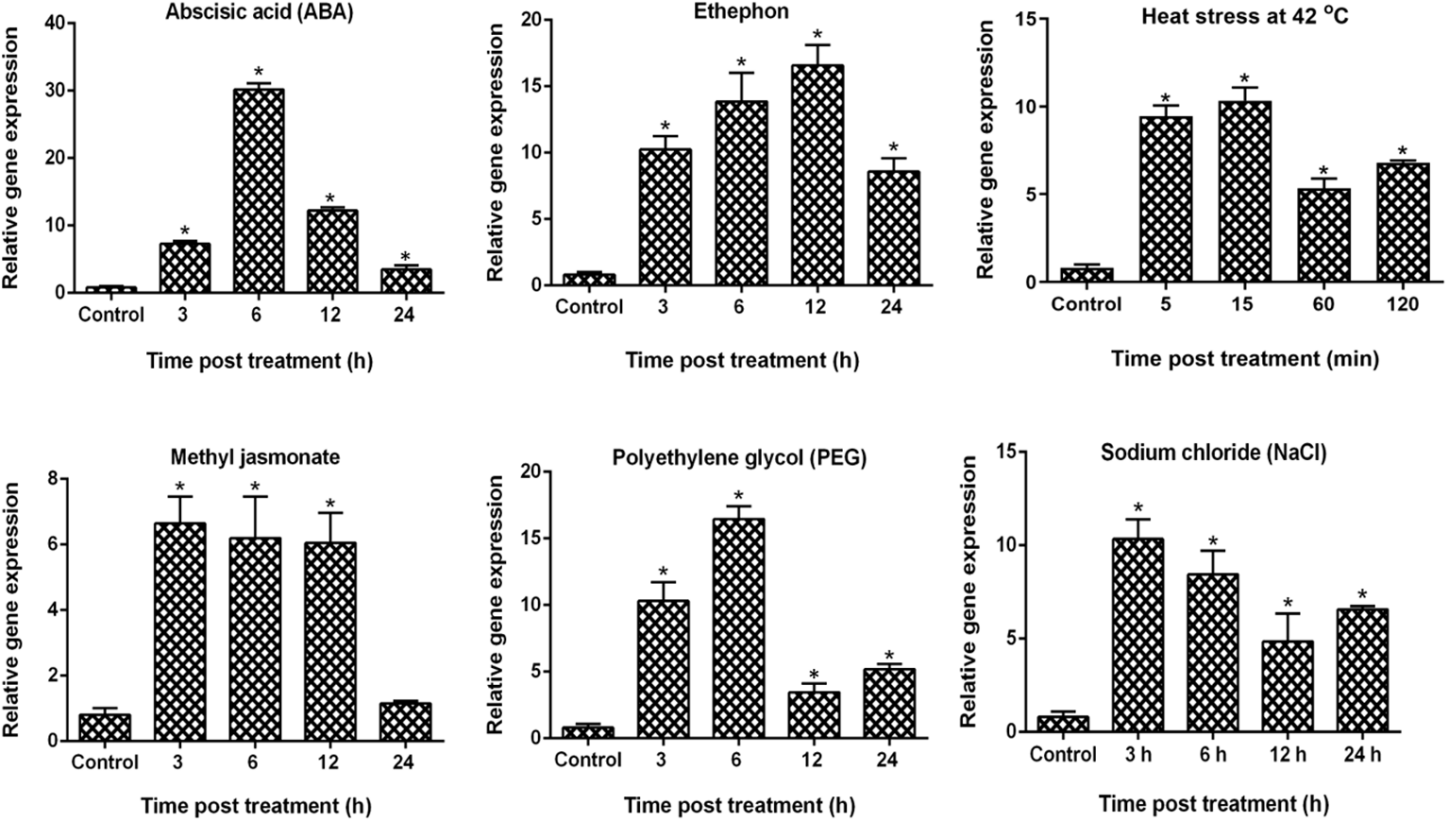
Relative expression analysis of *VaDJI* gene in *V. aconitifolia* by using qRT-PCR in response to various hormonal and abiotic stresses. Seedling samples collected at various time points were treated with PEG, heat stress, ABA, NaCl, ethephon and methyl jasmonate. Mean values of the fold change values of three biological and technical replicates for the expression study were used in the qRT-PCR analysis. Livak’s ΔΔC_T_ method was used to calculate the expression level. The experiment here included three biological and three technical repeats. Values are mean ± SE (* at p ≤ 0.05 value).

Hsp40s, also termed as J-domain proteins or DnaJ proteins constitute one of the important plants Hsps. The J proteins are categorized into Type-I, Type-II, and Type-III according to the presence or absence of the domains as depicted in **Supplementry Figure 1** (Liu and Whitham, 2013). Type-I DnaJ proteins usually contain J-domain (approximately 70 amino acid sequences), which interacts with Hsp70/DnaK (its bacterial counterpart), a proximal Gly/Phe-rich region (G/F), a zinc finger domain (CxxCxGxG) and a C terminal domain (less conserved) (Silver and Way, 1993; Craig et al., 2006). The occurrence of the HPD motif (histidine, proline and aspartic acid) is the hallmark of the J-domain and is crucial for the ATPase activity of Hsp70 and protein folding (Kampinga and Craig, 2010). Type-II proteins lack the zinc finger domain and the Type-III proteins exhibit only the J-domain whereas Type-IV (J like proteins) possess significantly similar sequences and structures like the J-domain, which lacks the HPD motif (Liu and Whitham, 2013). Pulido and Leister used the classification of DnaJA, DnaJB, DnaJC and DnaJD representing the four classes listed earlier (Pulido and Leister, 2018). Further, they have identified some novel DnaJ-like proteins classifying them as DnaJE and DnaJF proteins.

Molecular chaperones help avoid inappropriate protein association or aggregation of naked hydrophobic regions of unfolded or partly folded proteins and guide them for productive folding, transport and also promote non-productive interaction and aggregation with other proteins (Miernyk, 1999; Yang et al., 2010). Several studies on the role of Hsps report that some of the members are also induced by soil salinity, water, cold, high temperature and osmotic stresses in plants (Waters et al., 1996; Wang et al., 2004; Yang et al., 2010). Interestingly, plants DnaJ proteins have been reported to play diversified roles in physiological processes such as chloroplast movement (Suetsugu et al., 2005), flowering time (Park et al., 2011; Shen et al., 2011), and male sterility (Tamura et al., 2007; Liu and Whitham, 2013). In biotic stress responses, J proteins have been shown to interact with viral coat and movement proteins, thus facilitating viral assembly and the movement of viral particles during host-pathogen interactions (Shimizu et al., 2009; Liu and Whitham, 2013). A recent study showed that virulence effector HopI (class III J protein) from *Pseudomonas syringae* suppresses the SA (salicylic acid) accumulation and host defense responses in *Arabidopsis* plants (Jelenska et al., 2007). Several studies on the role of Hsps reported that some of its members are also induced by soil salinity, water, cold, high temperature and osmotic stresses in plants (Waters et al., 1996; Wang et al., 2004; Yang et al., 2010). In addition, overexpression of DnaJ (Type-III) from *Arachis diogoi* potentiated tolerance to multiple stresses in tobacco (Rampuria et al., 2018).

*Vigna aconitifolia* (moth bean) is a heat- and drought-tolerant legume crop grown in tropical, sub-tropical, and warm-temperate regions of various countries (Tiwari et al., 2018). This crop is an important biological resource to study the tolerance mechanisms due to its evolved morpho-physiological and gene-reservoir features for drought amelioration (Iseki et al., 2018). A previous study using the construction of suppression subtractive hybridization (SSH) cDNA library in the heat-tolerant genotype of *Vigna aconitifolia* RMO-40, (moth bean) identified the DnaJ-like protein to be induced upon treatment at an early stage of heat stress (within 5 min) (Rampuria et al., 2012). In line with this observation, the DnaJ family members appeared to be induced in pepper under heat stress conditions (Fan et al., 2017). A DnaJ protein is also reported to be induced in the thermotolerance of *Lentinula edodes* in combination with IAA metabolism (Wang et al., 2018b).

In the present study, we cloned this candidate gene from *Vigna* and analysis of its nucleotide and amino-acid sequences depicted that it is a DnaJ Type-I encoding gene, *VaDJI* exhibiting the zinc finger motif in its C-terminal domain. Overexpression of this gene in *Nicotiana benthamiana* suggested a pivotal role of *VaDJI* in ABA insensitivity and improved drought tolerance by modulating photosystem II efficiency and photosynthesis. The mechanism of drought tolerance due to *VaDJI* overexpression was outlined.

## 2 Materials and methods

### 2.1 Plant material

For cloning *VaDJI* gene, *Vigna aconitifolia* RMO-40 was used. The transformation was carried out in the tobacco *Nicotiana tabacum* (cv. Samsun) and the transgenic tobacco lines were used to perform molecular experiments in this study.

### 2.2 Hormonal and stress treatments of *Vigna aconitifolia*

Seeds of *Vigna aconitifolia* planted in pots filled with sterilized vermiculite were watered with 1x Hoagland’s solution. The seedlings were raised normally for seven days before subjecting to hormonal, salt and heat stress treatments (Harsh et al., 2016). The seedlings were kept in the relevant solutions for various chemical treatments. Treatments included NaCl (100 µM), methyl jasmonate (100 µM), abscisic acid (100 µM), ethephon (250 µM), heat stress at 42°C and polyethylene glycol (PEG-6000). Water was used as the mock for control.

### 2.3 Construct preparation and sequence analysis of *VaDJI* from *Vigna aconitifolia*

The full-length *VaDJI* CDS sequence was amplified from *Vigna aconitifolia* (RMO-40) using gene-specific primers listed in the earlier cDNA-AFLP study (Rampuria et al., 2012). The PCR product of *VaDJI* was cloned into a pRT100 vector expression cassette using *Apa*I and *Kpn*I restriction sites at the 5′/3′ end of the sequence (Gautam et al., 2019). The *VaDJI* cassette was excised from pRT100 using *Pst*I restriction digestion and it was then cloned into the appropriate site of the binary vector pCAMBIA2300. The recombinant pCAMBIA2300-*VaDJI*/35S-*nptII* construct contains CaMV35S (cauliflower mosaic virus) promoter and *nptII* as plant selectable marker. The recombinant construct was further mobilized into the *Agrobacterium* strain, LBA4404 and transformed into tobacco plants using the standard leaf disc method (Gallois and Marinho, 1995). Sequence analysis was done using SMART (http://smart.embl-heidelberg.de/), NCBI-CDD (https://www.ncbi.nlm.nih.gov/Structure/cdd/wrpsb.cgi) and the Phylogenetic trees were constructed using MEGA 7.0 software and ClustalW (https://www.genome.jp/tools-bin/clustalw), respectively to conduct multiple sequence alignment (Thompson et al., 1994; Kumar et al., 2016).

### 2.4 Plant transformation

Tobacco leaves from a one-month-old plant were surface sterilized in aqueous 0.1% mercuric chloride for 3 min. After rinsing five times in sterile distilled water, the leaf sections of 1.0 cm length were cut and incubated with *Agrobacterium tumefaciens* strain LBA4404 harboring the binary vector with the *VaDJI* cassette for 30 min in LB medium with the combination of antibiotics (25 mg/l each of Rifampicin and Kanamycin) at 28°C. The infected explants were further co-cultivated for 48 h on full-strength Murashige and Skoog (MS) culture medium having 2.0 mg/l 6-benzylaminopurine (BAP), 0.01 mg/l naphthalene acetic acid (NAA). After cocultivation, the explants were washed thrice for 5 min each in sterile water containing 250 mg/l Cefotaxime and then blotted dry on sterile filter paper. The *Agrobacterium*-treated leaf explants were kept on MS shoot regeneration medium (2 mg/l BAP + 0.1 mg/l NAA + 125 mg/l Kanamycin and 250 mg/l Cefotaxime) for two weeks. The induced shoots were transferred to shoot elongation medium (MS + 1.0 mg/l, BAP + 125 mg/l Kanamycin + 250 mg/l Cefotaxime). The elongated green shoots were excised and placed on rooting media (MS + 0.5 mg/l, NAA + 125 mg/l Kanamycin + 250 mg/l Cefotaxime) for rooting. The rooted plantlets were then transferred to the greenhouse for acclimatization in pots filled with soil and vermiculite mixed in a ratio of 4:1.

### 2.5 Selection of positive *VaDJI* tobacco transgenic plants

The three-week-old T_2_ transgenic tobacco plants were screened by PCR for the presence of various elements in the T-DNA region using *nptII* and *VaDJI* gene-specific forward and reverse primers (**Supplementary Table 1**). Semi-Q PCR was done using tobacco transgenic plants that were one week old (T_2_ generation) to determine the high and low expression *VaDJI* transgenic lines. To identify susceptible and resistant seedlings, the seeds from the confirmed T_1_ plants were germinated on MS medium supplemented with the selective drug Kanamycin (150 mg/l). Transgenic lines that remained green after Kanamycin selection and displayed a 3:1 segregation ratio (as determined by the χ^2^ test) were continued to T_2_ generation. Each resistant transgenic plant’s 60–110 seeds were plated on germination media (MS + Kanamycin), and 100% seed germination was utilized to determine homozygosity (Gautam et al., 2020). The seedlings that revealed Kanamycin sensitivity and lacked trans-gene amplification were classified as null segregants (NS) in the first generation. These sensitive segregants segregated from the primary transgenic plants served as negative controls in all experiments. The list of primers used in the *VaDJI* functional characterization is listed in **Supplementary Table 1**.

### 2.6 Seed germination assay

The seed germination analysis of NS and transgenic lines seeds of tobacco was performed according to the protocol as described in earlier reports of Ahmed et al. (2018). The seed germination assay was performed with approximately 100 seeds and the germination percentage was calculated on daily basis while taking the seedlings with green cotyledons into account. The seed germination percentage of *VaDJI* tobacco seeds was calculated using medium supplemented with half strength MS medium containing ABA (0, 7 µM and 10 µM) and PEG-6000 (0, 5% and 7%) using NS as control. To ensure data repeatability, the experiment was performed with three biological replicates

### 2.7 Seedling assay

The seedling assay of *VaDJI* transgenics plants was carried out with 10-days-old T_2_ seedlings on media containing various concentration of ABA (0, 7 µM and 10 µM) and PEG-6000 (0, 5% and 7%). To ensure data reproducibility, the experiment was performed with three biological replicates. Images of seedling morphology and measurements of root length were taken twelve-days after treatment.

### 2.8 Relative water content

Three-week-old seedlings grown on half strength MS medium were transferred to liquid half strength MS media enriched with ABA (0, 7 µM and 10 µM) and PEG-6000 (0, 5% and 7%) for 72 hours in order to determine the relative water content. The seedling fresh weight (W) was measured, and the samples were fully hydrated for 12 hours to yield a turgid weight (TW). To calculate the dry weight, samples were oven-dried for 48 hours at 60°C (DW). The following equation was used to calculate the relative water content.


RWC (%)=[(W−DW)/(TW−DW)]×100

### 2.9 Thermotolerance assay

The 15-day-old seedlings of NS and transgenic lines #4, #14, and #20 were transplanted and cultivated for three weeks in a growth chamber (Orbitek, Scigenics, Tamil Nadu, India) at 26°C, 70% humidity in plastic cups holding an identical weight of soil mixture (soilrite: soil = 1:4). The temperature of the growth chamber was gradually raised to 45°C for three hours and withering of the plantlets were recorded to evaluate thermotolerance (Dai et al., 2020).

### 2.10 Biochemical parameters

#### 2.10.1 Total chlorophyll and carotenoid estimation in transgenic plants

Ten seedlings from the control and treated samples in each trial were utilized for the biochemical estimation. The Hiscox and Israelstam technique was used to estimate the total chlorophyll and carotenoid levels using dimethyl sulfoxide (DMSO) (Hiscox and Israelstam, 1979).

#### 2.10.2 Lipid peroxidation and proline estimation in transgenic plants

Levels of lipid peroxidation were measured by determining the malondialdehyde (MDA) concentrations in control and stress-treated tissue samples using the TBARS (thiobarbituric acid reactive substances) method (Heath and Packer, 1968). The absorbance of the various samples was measured at 532 nm using a spectrophotometer (Shimazu spectrophotometer UV-1800, U.S.A.). Proline was extracted using the procedure outlined by Bates et al. (1973), and spectrophotometric measurements were made at 520 nm.

### 2.11 Tobacco abiotic stress survival assay

Control NS and tobacco plants overexpressing *VaDJI* were grown under an adequate watering regime for eight weeks in a greenhouse in pots with soil prepared using a mixture of soil: soil rite in a 4:1 ratio before being exposed to drought stress for fourteen days. During the stress treatment period, water to the plants was stopped to put them under drought stress. When dehydration in tobacco plants became fatal, watering in pots was resumed, and the plants were left to recover for three days. After drought treatment and rewatering, the pictures were used for analysis.

### 2.12 Staining with DAB (3, 3′-diaminobenzidine)

The *in-vivo* generation of H_2_O_2_ in the leaf tissue samples under stress treatments was determined by histochemical staining with diaminobenzidine (DAB) using leaf discs with a diameter of 0.5 cm that was collected from the stressed and control samples grown in pots. DAB was quantified by the procedure described by Wei et al. (2017).

### 2.13 Photosynthetic efficiency and chlorophyll *a* fluorescence indices

To monitor the leaf gas exchange in the leaves of control and drought stress-treated plants, a portable LI-6400XT infrared gas exchange equipment was used (Li-COR Inc, Lincoln, NE, USA). Using a broad leaf chamber equipped with PAR (photosynthetically active radiation, LCpro-32070) and a leaf probe, the gas exchange parameters *P_n_* (net photosynthetic rate; mol m^-2^ s^-1^), *g_s_* (stomatal conductance m^-2^ s^-1^), and *E* (transpiration rate; mmol m^-2^ s^-1^) of NS and transgenic plants were measured (ADC, M PLC-011). WUE (water usage efficiency, mol CO_2_ mol^-1^ H_2_O) was computed using the aforementioned information as *P_n_/E*.

A portable plant efficiency analyzer (Handy–PEA; Hansatech Instruments Ltd, Norfolk, UK) was used to record chlorophyll *a* fluorescence metrics such as *PI_abs_* (performance index) and *F_v_/F_m_* (maximum quantum yield). The measurements were taken after 30 min of the dark-adapted fully expanded third leaf of control and stressed plants that had been treated with drought stress. The data in the graph are the mean of three separate replications.

### 2.14 Quantitative RT-PCR of stress marker genes

Following the manufacturer’s instructions, total RNA was extracted from control and treatment samples using Tri-reagent (Takara, Biotech, UK) and further treated with RNase-free DNase1. First-strand cDNA was created using Prime-script First Strand cDNA Synthesis Kit (Takara, Biotech, UK) and 2 µg of total RNA. The cDNA was diluted 2.5 times (1:2.5) and 1 µl of diluted cDNA was used in a total reaction volume of 10 µl containing 10 µM of each primer, 2× Fast start SYBR green, milli-Q water for the analysis using quantitative reverse transcription PCR (Gautam et al., 2019).

The expression patterns of genes like *SOS1*, *SAMDC*, *ERF5*, *ERD10*, *APX*, *MnSOD*, *DREB3*, *ERF5*, *ERD10*, and *P5CS1* that have been shown in previous studies to be involved in the instantaneous response to abiotic stress in plants were analyzed to determine whether the ectopic expression of *VaDJI* is linked to the enhanced expression pattern of various above abiotic stress-specific genes. For the expression analysis by qRT-PCR, samples were collected as three biological replicates, and the *Ubiquitin* and *ADH3* genes were employed as internal controls. Relative expression patterns were determined using the 2^-ΔΔCT^ method (Livak and Schmittgen, 2001).

### 2.15 Statistical significance

The graphs for each experiment were created using the Graphpad Prism program, and one-way ANOVA with DMRT (Duncan’s multiple range test) was used to statistically analyze each data set. A single asterisk (*) indicates that the statistical difference was significant at the 0.05 level (p ≤ 0.05) and a double asterisk (**) is significant at the 0.01 level (p ≤ 0.01).

## 3 Results

### 3.1 Cloning and isolation of cDNA sequence of *VaDJI* from *Vigna aconitifolia*

The ORF of VaDJI protein encodes a 418 amino acid polypeptide along with a predicted molecular weight of 46.47 kDa and *pI* (isoelectric point) of 5.80. Sequence analysis using SMART, NCBI-CDD and phylogenetic study has led to its identification as Type-I DnaJ protein having the N-terminal J-domain consisting of HPD tripeptide, which is highly conserved, followed by G/F- rich region (Gly/Phe domain) and the DIF motif (**Supplementary Figure 2**). The ZBD (zinc-binding domain) displayed three quintessential CXXCXGXG motifs and a fourth motif ending in N instead of G (**Supplementary Figure 2**).

The amino acid sequences of DnaJ proteins from various species were used to align the deduced amino acids of VaDJI. VaDJI protein exhibits a maximum similarity of 98.80% to DJI protein from *Vigna radiata*. It exhibited similarities to the DJI proteins from *Glycine max* and *Phaseolus vulgaris*, (97.61 and 96.41% respectively) (**Supplementary Figure 3**). Interestingly, *Nicotiana tabacum* genome was found to carry three different isoforms of *DnaJI* i.e., *NtDJI*-1, *NtDJI*-2 and *NtDJI*-3. These proteins had a sequence similarity of 28%, 29% and 25% with *VaDJI*. VaDJI phylogenetic relationship revealed that it is closely linked to the *Vigna radiata* and *Phaseolus vulgaris,* both having 418-amino acid as opposed to *Glycine max* DnaJ proteins that carried a 417 amino-acids sequence (**Supplementary Figure 4**). The fact that all three plants are members of the Fabaceae family may account for their striking similarity in the amino acid sequences.

### 3.2 *VaDJI* expression analysis in response to various hormonal and environmental stress treatments

To examine the expression patterns of *VaDJI* in the *Vigna aconitifolia* RMO-40 variety in response to abiotic stress (heat stress and PEG) and phytohormonal treatments (ABA, MeJa and ethephon), we carried out qRT-PCR analysis using RNA samples collected at different time intervals (**Figure 1**). As displayed in **Figure 1**, *VaDJI* transcripts rapidly increased by the various treatments. The transcript abundance was strongly elevated with 6 h of ABA and PEG treatment followed by ethephon at 12 h of treatment. *VaDJI* transcripts recorded significant early upregulation up to 11 and 10 fold for PEG and heat treatment at 3 h and 5 min, which increased at 6 h and 15 min respectively. Interestingly, the observed expression was 4 fold and 5 fold higher than the basal expression level with PEG stress at 12 and 24 h of treatment. A similar pattern of transcript accumulation was evidenced during heat stress where the increment of expression was approximately 5 to 7 fold higher than that of the basal level at 1 and 2 h of treatment.

We observed that MeJa treatment also caused a significant increase in transcript level initially during 3 h, and then further decrease at 6 and 12 h followed by 5 fold decrease at 24 h of treatment. In response to ethephon treatment, *VaDJI* expression was relatively elevated from 3 to 12 h (10 to 17 fold), with a decline at 24 h up to 8 fold (**Figure 1**).

ABA is a key signalling molecule in plants in regulating abiotic stress tolerance. Under the ABA treatment, we found that there was an early induction of *VaDJI* transcripts to nearly 8 fold at 3 h, followed by a 31 fold increment at 6 h, which declined gradually (**Figure 1**). Strong upregulation was noticed under ABA treatment as compared with the other phytohormones suggests a possibility that ABA serves as an important regulator of *VaDJI* expression. Overall, upregulation of the *VaDJI* during the above treatments, indicated it to be involved in various interconnected signalling pathways controlled by these signalling molecules.

### 3.3 Screening and molecular characterization of *VaDJI* expressing tobacco transgenic plants

**Figure 2:**
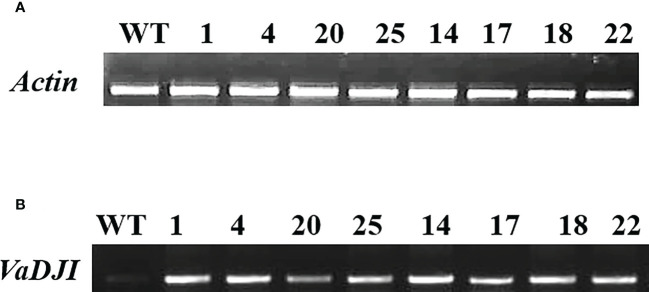
Semi-Quantitative real-time analysis of *VaDJI* T_2_ transgenic lines using specific primers for *VaDJI* gene and *actin* (as an internal reference). **(A, B)**- Lane1: represents cDNA from wild type (WT). Lane 2-9: represents cDNA from transformants. Depending on the level of expression, lines L-20, L-25 and L-17 were considered low expression lines. Lines L-1, L-4, L-14 and L-18 were considered high expression lines.

Several three-week-old T_2_ transgenic tobacco plants grown on Kanamycin selection overexpressing a cDNA cassette of *VaDJI (*pCAMBIA2300-*VaDJI/*35S-*nptII)* were screened by PCR using *npt*II and *VaDJI* gene specific primers. The transgenic plants displayed the presence of 564 bp and 1.27 kb corresponding to *nptII* and *VaDJI* respectively, whereas the negative control NS plants did not exhibit the presence of any amplification (**Supplementary Figures 5A, B**). The transgenic plantlets were screened by semi-quantitative RT-PCR to identify the high and low expression lines among the transgenic plants (**Supplementary Figures 6A, B** and **Figures 2A, B**). Based on the band intensity of semi-quantitative RT-PCR analysis, lines L-20, L-25 and L-17 were considered low expression lines whereas Line L-1, L-4, L-14, L-18 and L-22 were considered as high expression lines (**Figures 2A, B**).

### 3.4 Overexpression of *VaDJI* led to insensitivity to exogenously applied ABA during germination and seedling growth stage

**Figure 3:**
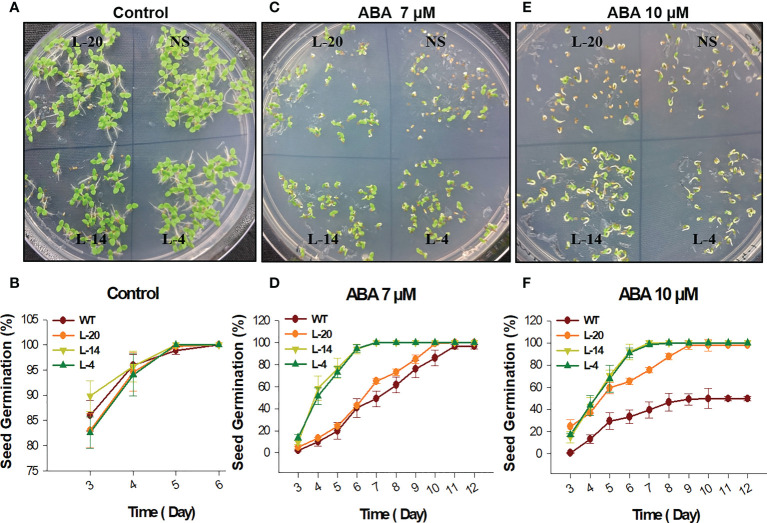
Seed germination analysis of *VaDJI* transgenic lines for ABA insensitivity; **(A, C, E)** Represents phenotypic difference during control, 7 µM ABA and 10 µM ABA treatments, **(B, D, F)** germination percentage of NS and *VaDJI* transgenic lines under control, 7 µM ABA and 10 µM ABA stresses. Experiments were repeated with three technical and three biological repeats. Error bar depicts mean ± S.E.

The phytohormone ABA is considered to be a major mediator of abiotic stresses and plant development (Vishwakarma et al., 2017; Miao et al., 2018; Chen et al., 2020). Since *VaDJI* expression was strongly upregulated during ABA treatment, we examined whether *VaDJI* participates in the ABA-mediated reduction of seed germination and/or seedling growth stage by observing the seed germination percentage in NS and transgenic lines in presence of exogenous ABA at the different concentrations on daily basis (0, 7 µM and 10 µM) (**Figures 3A–F**). In presence of 7 µM and 10 µM ABA, we observed severe inhibition in the pattern of seed germination of NS lines compared (**Figures 3C–F**) with the ABA-free medium where a similar pattern of seed germination was clearly visible in all the lines (**Figures 3A, B**). At the end of the 6^th^ day of 10 µM ABA treatment, only 33.3 ± 6.0 of NS seeds showed germination as compared to 60- 98.6% of seed germination rate in transgenic lines **(** **Figure 3F**).

**Figure 4:**
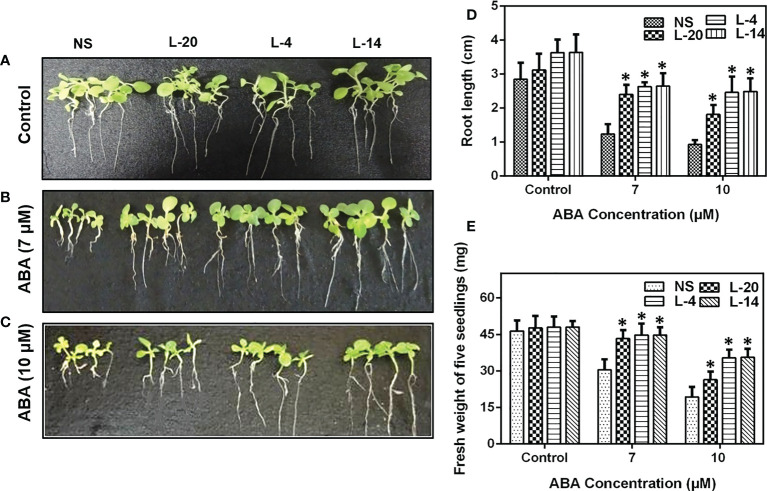
Analysis of enhanced ABA insensitivity in transgenic lines of *VaDJI*; **(A)** Under control condition, **(B)** 7 µM ABA, **(C)** 10 µM ABA. Photographs of NS and transgenic lines were taken after giving exogenous ABA stress for 12 days of growth. The difference observed in the seedlings **(D)** root length, **(E)** fresh weight in the presence of 7 µM ABA and 10 µM ABA. Data were represented as mean ± SE. Statistical significance was tested using one-way analysis of variance (ANOVA) and *(single asterisk) indicates p ≤ 0.05.

We then checked the ABA insensitivity at the seedling stage of these lines. We found that seven-day-old *VaDJI* expressing T_2_ tobacco lines L-4, L-14 and L-20 showed an increase in root length when moved to media containing 7 µM ABA whereas the NS lines continued to maintain the length of roots under ABA free medium (control condition) **(** **Figures 4A–D**). As the concentration of ABA increased from 7 µM to 10 µM, root length and fresh weight drastically reduced in NS against the transgenic lines which exhibited longer roots, depicting that the seedlings are ABA insensitive (**Figures 4C–E**).

### 3.5 *VaDJI* tobacco lines showed enhanced tolerance under heat stress

To study the role of *VaDJI* under heat stress five-week-old plants of NS and transgenic lines L-4, L-14, and L-20 were exposed to 45°C in a growth chamber. The high expression lines L-4 and L-14 remained healthy while the NS plants displayed signs of severe withering and chlorosis, a sign of heat damage after 2 hours and were fully wilted after 3 hours of treatment (**Supplementary Figures 7A, B**). The top crown of leaves of low expression line L-20 also appeared robust though it showed some signs of wilting (**Supplementary Figures 7A, B**).

### 3.6 Drought stress tolerance of seedlings harboring *VaDJI*

**Figure 5:**
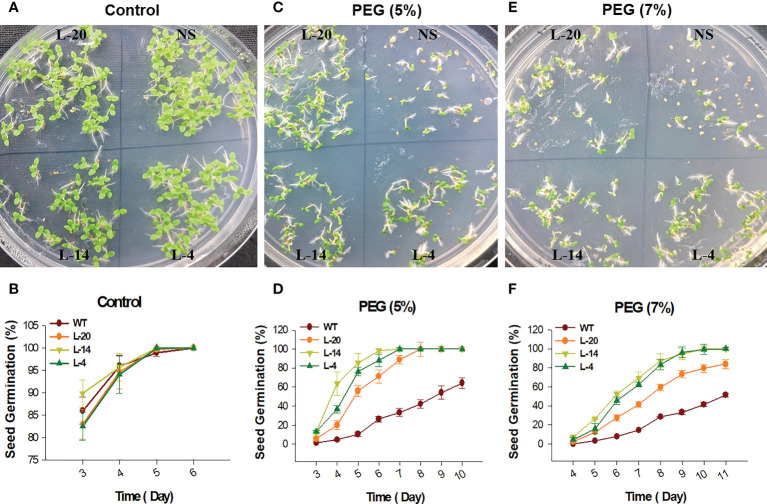
Seed germination analysis for PEG stresses tolerance; **(A, C, E)** Represents phenotypic difference during control, and PEG treatments, **(B, D, F)** germination percentage of NS and *VaDJI* transgenic lines under mock, 5% PEG and 7% PEG stress. Experiments were repeated with three technical and three biological repeats. Error bar depicts mean ± S.E. Statistical significance was tested using one-way analysis of variance (ANOVA) and *(single asterisk) indicates p ≤ 0.05.

**Figure 6:**
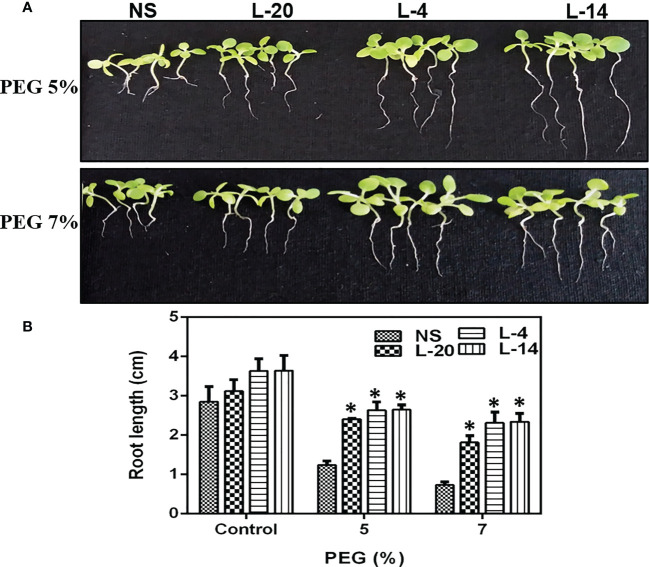
Analysis of enhanced PEG endurance in transgenic lines of *VaDJI*; **(A)** under 5% PEG, and 7% PEG. Photographs of NS and transgenic lines were taken after drought stress for 12 days of growth., **(B)** Difference observed in the seedlings root growth in the presence of 5% and 7% PEG 6000. Data were represented as mean ± SE. Statistical significance was tested using one-way analysis of variance (ANOVA) and *(single asterisk) indicates p ≤ 0.05.

The T_2_ generation transgenic lines of *VaDJI* were subjected to plate-based using PEG that simulates drought stress. The seeds used in the tests were consistently 100% germinated from transgenic lines on a seed germination medium containing Kanamycin. Seed germination rates for the NS and *VaDJI* lines were nearly identical in the absence of a stress media. We observed that a rise in the PEG concentration from 5% to 7% in the germination medium had a significantly negative impact on the germination percentage of NS seeds (**Figures 5A–F**). Also, compared to NS plants, transgenic seedlings grew more rapidly when exposed to abiotic stress. Significant variations in root length between *VaDJI* expressing and NS seedlings were seen as PEG concentration increased from 5% to 7% (**Figures 6A, B**). During PEG stress, the transgenic lines significantly increased the length of their roots compared to NS plants by 50-80%.

### 3.7 Overexpression of *VaDJI* resulted in increased drought tolerance

**Figure 7:**
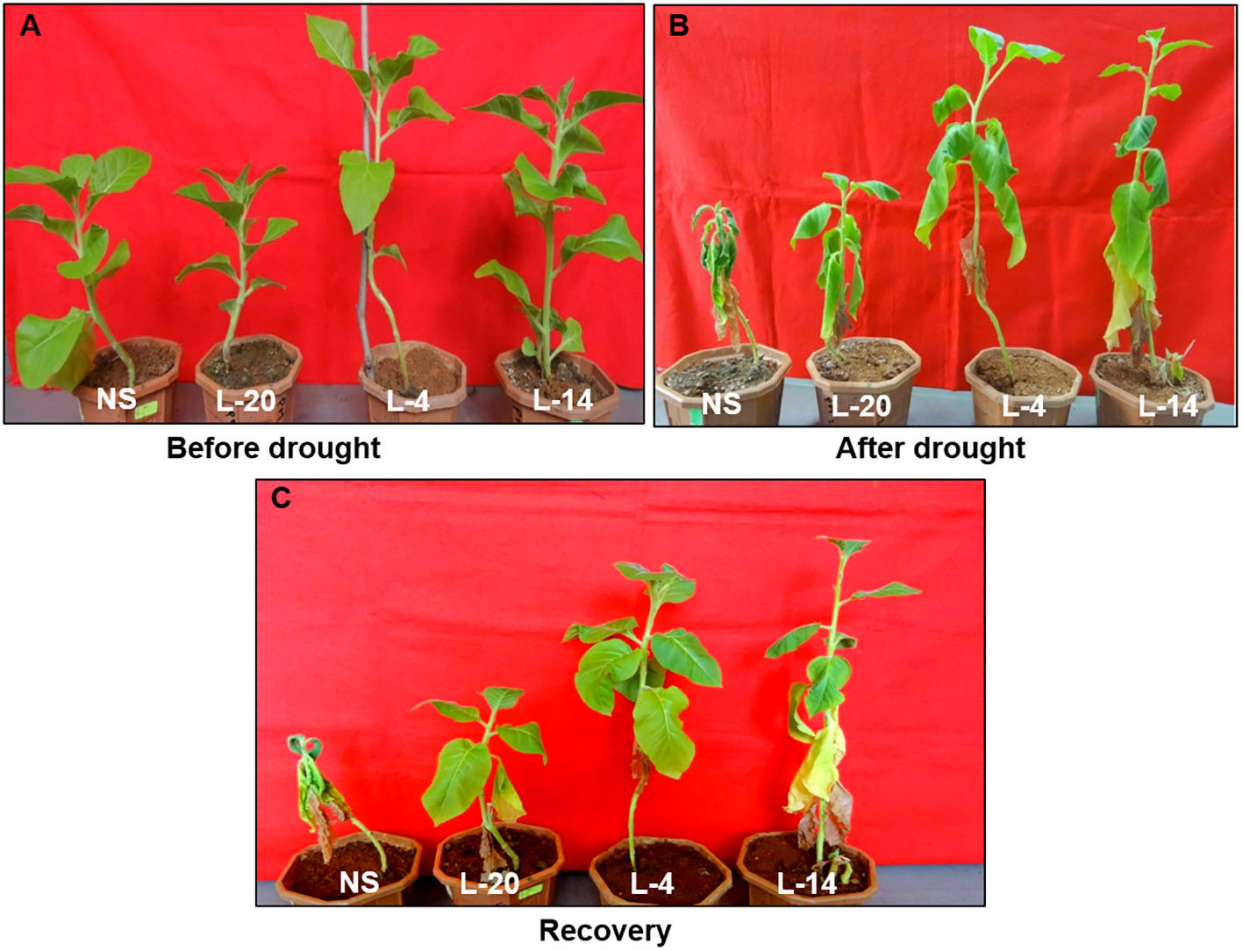
Assessment of drought tolerance observed in eight week NS and *VaDJI* transgenic tobacco plants subjected to drought stress at pot level; **(A)** Under control condition, **(B)** After 14 days of drought stress, **(C)** recovery phase after drought.

To explore whether *VaDJI* overexpression could alter the drought responses in transgenic tobacco plants, we investigated the performance of *VaDJI* and NS plants under drought stress in pots. The *VaDJI* transgenic lines after drought stress exhibited reduced wilting, growth inhibition and curling of leaves compared with NS lines. Moreover, NS lines also displayed retarded growth followed by chlorosis and wilting (**Figures 7A, B**). Interestingly, the tobacco transgenic lines after resuming the supply of water demonstrated complete recovery of the normal phenotype with the presence of healthy leaves, while the NS plant showed wilting and bleached symptoms even after three days of rewatering conditions (**Figure 7C**).

### 3.8 Drought tolerance of *VaDJI* overexpression lines was associated with increased water retention, higher proline and reduced MDA levels

**Figure 8:**
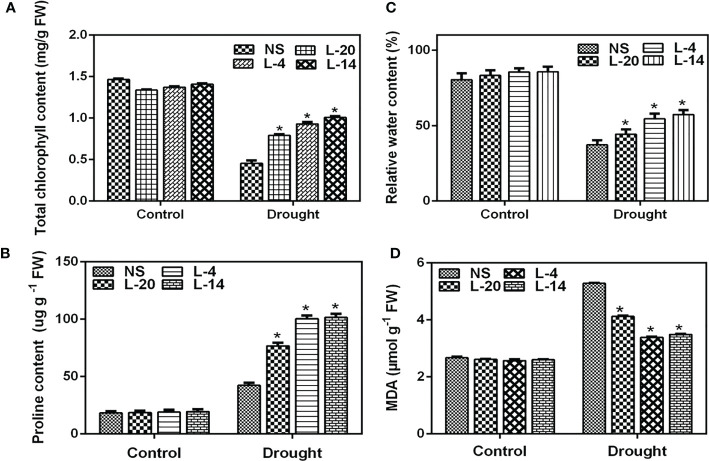
Effect of drought treatment on tobacco transgenics harboring *VaDJI* after 14 days of drought stress; **(A)** Estimation of total chlorophyll content, **(B)** Proline content, **(C)** RWC relative water content, **(D)** MDA levels in NS and transgenic tobacco lines. Data were represented as mean ± SE. Statistical significance was tested using one-way analysis of variance (ANOVA) and *(single asterisk) indicates p ≤ 0.05.

Further examination of the relative water content (RWC), proline and MDA levels depicted that these parameters recorded significant differences between NS and high expression lines of *VaDJI*. RWC is an appropriate tool for measuring drought tolerance and also determining the plant water status whereas total chlorophyll represents the level of chlorosis. RWC, proline and total chlorophyll contents of the transgenic lines were significantly higher than compared to NS lines after they were exposed to fourteen days of drought stress (**Figures 8A–C** **)**. Under control conditions, the transgenic lines and NS did not show any significant variation in MDA content. Whereas, MDA levels were significantly lower in the transgenic tobacco relative to NS lines under drought stress, suggesting reduced membrane damage (**Figure 8D**). The leaves of NS and transgenic lines stained by DAB under standard growing conditions displayed a similar pattern of DAB staining. But following drought stress, NS leaves displayed more vivid and profound brown patterns in bleached leaves in comparison to the *VaDJI* overexpressing lines, and H_2_O_2_ buildup was noticeably higher in the NS (**Supplementary Figures 8A–C**). These physiological parameters established that the *VaDJI* transgenic plants were more tolerant to drought stress.

### 3.9 *VaDJI* alleviates photosynthetic performance and photoinhibition of photosystem II

**Figure 9:**
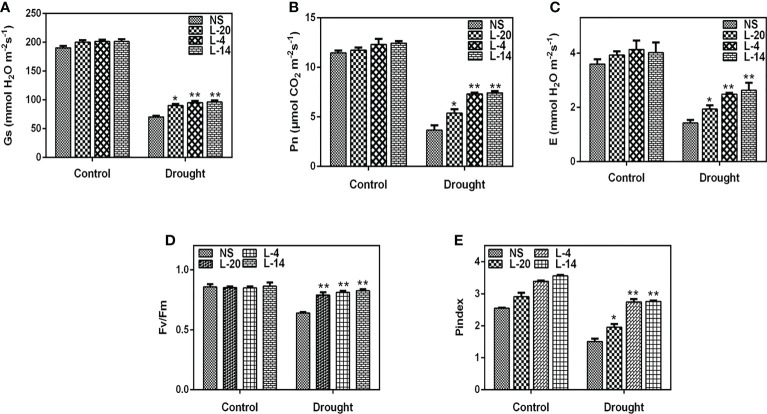
Effect of drought treatment on leaf gas exchange parameter and chlorophyll fluorescence of tobacco transgenics harboring *VaDJI* after 14 days of drought stress; **(A)** *g_s_* – measurement of stomatal conductance, **(B)** *P_n_* - measurement of net photosynthetic rate), **(C)** E- measurement of transpiration rate, **(D)** F_v_/F_m_ - measurement of PSII photochemistry efficiency, **(E)** PI_abs_- measurement of performance index. The values indicate mean ± SE. *statistically significantly different at p ≤.05 and **statistically significantly different at p ≤ 0.01 level as compared to NS.

The reduced photosynthetic efficiency and the production of ROS (reactive oxygen species) are believed to be significant variables that influence plant performance under environmental stress conditions (Huang et al., 2019; Dumanović et al., 2021). Therefore, photosynthetic traits and chlorophyll *a* fluorescence in null segregants and transgenic plants were evaluated in order to comprehend the effects of drought stress on photosynthesis. We have observed that when tobacco lines were exposed to drought stress, the three transgenic lines viz., L-20, L-4 and L-14 were found to have considerably higher *P_n_* (net photosynthetic rate) and *g_s_* (transpiration rate) than those of the NS lines (**Figures 9A, B**). Similar findings were also made while measuring stomatal conductance *E*, where the drop in NS was more pronounced than in transgenic lines, especially lines L-4 and L-14 (**Figure 9C**). The *F_v_/F_m_* ratios, which elaborate PSII photochemistry efficiency in all three transgenic lines was similar in well-watered conditions (0.86 to 0.87) and NS (0.85), whereas the high expression lines L-4 and L-14 showed a quantum efficiency of 0.82 to 0.83 under drought stress, which was very similar to the control conditions. Compared to the low expression line L-20, the NS plants displayed a quantum efficiency value of 0.71 (**Figure 9D**). This demonstrates unequivocally that the photosynthetic efficiency decreased in the drought-stressed NS plants and was much lower than in the *VaDJI* overexpressing lines. Interestingly, the performance index (*PI_abs_*) in the NS lines dropped by 48% whereas the best *VaDJI* transgenic lines (L-4 and L-14) only showed a decline of 11–15% and the low expression line (L-20) only showed a decline of 34% (**Figure 9E**).

### 3.10 *VaDJI* regulates stress markers and ROS-related genes under drought treatment

**Figure 10:**
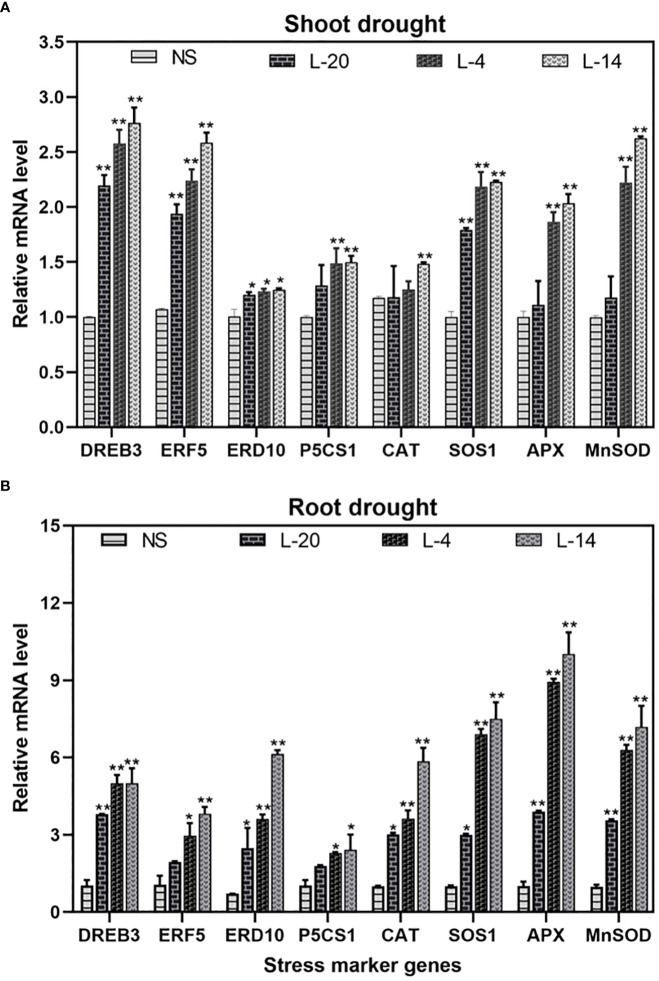
Relative expression analysis of stress- marker genes in **(A)** Shoot **(B)** Root of NS and *VaDJI* transgenic tobacco plants by using qRT-PCR. Plants of tobacco after 14 days of drought stress were used for the expression analysis. Relative expression was normalized with the mean of *ADH3* and *UBI* genes as endogenous control. Livak and Schmittgen’s ΔΔC_T_ method was used to calculate the expression level. The experiment here included three biological and three technical repeats. Values are mean ± SE (*at p ≤ 0.05 value and **at p ≤ 0.01).

To achieve deeper insight into *VaDJI* role in conferring drought tolerance, expression analysis of stress markers and ROS-related genes under untreated and drought conditions in shoot and root tissues was studied. We found that the transcript levels of *DREB3* (*Dehydration-responsive element-binding protein*), *ERF5* (*ethylene response factor*), *ERD10* (*early response to dehydration*), *P5CS1* (*Δ1-pyrroline-5-carboxylate synthetase*), *SOS1* (*salt overly sensitive*) genes were higher in *VaDJI* tobacco lines even in control conditions. The transcript levels of the ROS-detoxification-related genes such as *APX*, *SOD* and *CAT* also showed a significant increase in expression in transgenic lines compared with the NS plants under drought stress (**Figures 10A, B**). The relative expression analysis of stress-marker and ROS-related genes revealed that *VaDJI* overexpression enhanced drought tolerance in transgenics as compared to NS plants.

## 4 Discussion

The studied gene *VaDJI* belongs to Hsp40, a member of type I J-proteins family and these proteins work in association with the Hsp70 proteins function inprotein folding, refolding and transport as well as removal of the damaged cellular proteins. Hsp40 type proteins interact with the Hsp70 proteins through the conserved J-domain present in them. They add specificity to the Hsp70 protein function through their variable domains, which respond to environmental cues. Unfavourable climatic conditions cause an increase in ROS levels above threshold values, which hampers plant physiology and resulting in an increase amount of non-functional proteins in cells compromising the protein cellular homeostasis. All these factors result in restricting plant growth and development. The cellular protein homeostasis is modulated and maintained by the cellular chaperone machinery to alleviate the damage caused to the cellular proteins by abiotic stresses. Therefore, the expression of stress-responsive genes that code for the molecular chaperones like heat shock proteins is one of the tolerance mechanisms to protect plant cellular components and restore cellular homeostasis (Salas-Muñoz et al., 2016).

In plants, limited published information is available on the characterization of the Dna J proteins belonging to Type I class in abiotic stress tolerance. *Arabidopsis AtDjB1*, a Dna J encoding gene facilitated thermotolerance by shielding the plants against oxidative damage induced by heat stress (Zhou et al., 2012). Similarly, *AtDnaJ* overexpression also conferred NaCl tolerance in transgenic *Arabidopsis* lines (Zhichang et al., 2010). However, there is no published evidence on the involvement of Type-I DnaJ proteins of the heat tolerant plant *Vigna aconitifolia* in response to drought stress so far. Here, we report that ectopic expression of *VaDJI* conferred ABA insensitivity at the seedling level and drought endurance at vegetative growth stages.

In the current study, *VaDJI* was isolated from *Vigna* using an SSR cDNA library and was found to be induced by signalling molecules and abiotic stress treatments (Rampuria et al., 2012). DnaJ proteins are known to be induced by heat, intense light, cold, methyl viologen (MV) and to play roles in signal transduction, development and stress tolerance (Xia et al., 2014; Lee et al., 2018; Rampuria et al., 2018; Wang et al., 2018a; Wang et al., 2019a; Wang et al., 2020; Li et al., 2021). In the present study, *VaDJI* is rapidly upregulated in response to all the stresses such as MV, ethephon, heat stress and PEG treatments. This early response to a wide range of stimuli suggests that *VaDJI* plays an important role in arbitrating various stress responses in plants. Being a co-chaperone that works in association with Hsp70, this DnaJ gene may be required for the proper folding of different nascent stress-responsive proteins when they encounter stressful situations.

Emerging evidence indicates that ABA is a crucial plant hormone involved in controlling stress responses and also minimizing water loss. During drought stress conditions, ABA modifies ion transport in guard cells by encouraging stomatal closure and blocking stomatal opening (Sah et al., 2016; Rehman et al., 2021). In our present study, various observations shed light on the function of *VaDJI* in plants under drought conditions. Our observations suggest that the ABA-driven thermotolerance response in tobacco may be mediated by *VaDJI*. This interpretation is influenced by following factors: First, qPCR results showed that heat and ABA treatment rapidly elevated the *VaDJI* transcript levels. Second, the overexpression of *VaDJI* resulted in drought tolerance and ABA insensitivity at the seed germination and seedling stage. Several studies have previously reported that the constitutive expression of specific genes viz., *WRKY20*, *GmbZIP62 OsPP108*, and *ABI5* confers ABA insensitivity during the seed germination stage along with abiotic stress tolerance such as drought, salt, and alkalinity (Brocard et al., 2002; Liao et al., 2008; Luo et al., 2013; Singh et al., 2015; Ahmed et al., 2018; Collin et al., 2020). These findings are consistent with present study and suggested that ABA signalling controls seed germination as well as provided resistance to abiotic stimuli through a variety of pathways.

Drought tolerance in plants generally involves drought escape (by short life span or developmental plasticity of the plants), drought avoidance (by reduced water loss and enhanced water uptake in plants) and drought tolerance (by antioxidant capacity, osmotic adjustment and desiccation tolerance) (Yıldırım and Kaya, 2017; Polle et al., 2019). Interestingly, in this study, overexpression of the *VaDJI* gene in tobacco remarkably improved root growth, plant water status and better solute accumulation under drought stress. Most notably, the transgenic lines showed normal growth, despite the fact that plant height was increased compared to NS lines even under normal conditions and phenotypic differences between them further increased under drought stress. It is assumed that *VaDJI* gene alters the developmental processes by affecting the phytohormone status i.e., auxin and cytokinin levels in the plant. It has been reported that *ARG1* (altered response to gravity) gene encoding a *DnaJ* like protein in *Arabidopsis* which has a role in signalling pathways related with gravity and also interact with the cytoskeleton (Sedbrook et al., 1999). It is possible that *VaDJI* may alter growth in tobacco transgenic plants in a way similar to *ARG1* by affecting the cytoskeleton since mutations in the *ARG1* locus affected not only root and hypocotyl growth responses to plant hormones but also auxin transport inhibitors and accumulation of starch. *ANGULATA7* encodes a *DnaJ* like protein that is engaged in organization of thylakoidal membrane and leaf development (Muñoz-Nortes et al., 2017). In our study, *VaDJI* gene promoted the growth of transgenic root system with a in biomass of transgenic lines compared to NS lines. This suggests that tobacco plants can alter organ growth for water uptake to counter the limited water conditions resulting in higher plant water status. Moreover, the promoted vegetative growth is further supported by the comparable chlorophyll levels of the transgenic plants compared with the NS plants.

We have also shown that the better stress endurance of transgenic plants is related with lower MDA content and enhanced accumulation of solutes. MDA represents lipid peroxidation triggered by ROS and is generally used as an indicator of damages mediated by ROS in plants (Moore and Roberts, 1998). Hence, this study signifies that lipid peroxidation mediated by ROS injuries is alleviated in the *VaDJI* transgenics under drought stress. Proline acts as an osmolyte that assists in protein stability and scavenging of free radicals (Szabados and Savouré, 2010). In comparison with the NS plants, transgenic lines displayed almost similar proline content but the difference in proline accumulation under drought stress increased significantly. Also, the overexpression of *P5CS*, one of the key enzymes catalyzing the proline biosynthesis pathway has been reported to enhance stress tolerance in plants (Lohani et al., 2022; Ma et al., 2022). For instance, overexpression of *P5CS* genes in *Cajanus cajan*, *Oryzae sativa* and *Solanum tuberosum* enhanced salt tolerance in the corresponding transgenic plants (Hmida-Sayari et al., 2005; Kumar et al., 2010; Surekha et al., 2014).

The primary component determining the composition of crop productivity is photosynthesis, which serves as the foundation for crop growth and development (Richards, 2000; Fang et al., 2018). Drought stress can negatively affect photosynthetic capacity, denature proteins, increase ROS production, and create a metabolic imbalance (Zhang et al., 2022b). Interestingly, the *VaDJI* transgenics outperform NS lines in showing strong chlorophyll fluorescence and improved *P_n_*, *g_s_*, *E*, and *WUE* values under drought stress. The relationship between improved photosynthetic performance and the expression of *VaDJI* is consistent with earlier reports in which the expression of *LeCDJ2* improved the efficiency of photosystem II and net photosynthetic rate (Wang et al., 2014). Similarly, *Arabidopsis* triple knock-down lines of M-sHSPs (mitochondrial small heat shock proteins) had altered levels of proteins that were mostly involved in photosynthesis and antioxidant defense. Also, in the knock-down plants, heat stress resulted in an unusual pattern of cytosolic response as well as the overexpression of other *sHSP* members. Overall, the loss of all three M-sHSPs in *Arabidopsis* had a significant negative impact on core metabolic activities, altering how the plant should grow and develop (Escobar et al., 2021). A pathogen-induced *AdDjSKI* (DnaJ protein) from a wild peanut *Arachis diogoi* enhanced the ability of tobacco and *E. coli* to withstand a variety of stresses (Rampuria et al., 2018). Ectopic expression of the Hsp40 gene (*Zjdjb1*) of *Zostera japonica* also enhanced thermotolerance in *Arabidopsis* (Chen and Qiu, 2022). These results are in line with the present study where transgenic lines exhibited better performance under multiple stress encounters in terms of growth and development as compared to NS.

To further gain an insight into the molecular mechanism of *VaDJI* in drought stress, the expression of ROS-detoxifying and stress marker genes was explored. The antioxidant system involving *SOD* provides the first lines of defense in countering the toxic ROS levels by catalyzing the conversion of O_2_^-^ to oxygen and hydrogen peroxide, of which the latter is further scavenged by combined actions of *CAT* and *POD* (Blokhina et al., 2003; Huang et al., 2010; Hu et al., 2013). Present observations demonstrated that the *VaDJI* transgenic lines recorded significantly higher expression of *CAT* and *SOD* genes compared to the NS lines. In the *VaDJI* transgenic plants, we noticed increased transcript levels of *MnSOD* and *APX* which are involved in ROS detoxification. In addition, we observed increased expression of stress marker genes such as *ERD10*, *DREB3*, *P5CS1*, and *SOS1* in the transgenic lines. Existing literature suggests that these genes are involved in mitigating plant stress tolerance in plants. *ERD10*, a member of the dehydrin family acts as a chaperone to shield plants from external stressors and promotes seed development (Kim and Nam, 2010; Gautam et al., 2020). *SOS1* encodes a Na^+^/H^+^ antiporter that regulates ion transport across the membranes and contributes to salt tolerance (Xie et al., 2022; Zhang et al., 2022a). *DREB3*, a transcription factor controls the stress responses in plants by interacting with the DRE/CRT *cis*-elements found in the promoter regions of stress-responsive genes (Shavrukov et al., 2016; Wang et al., 2019b). Based on these results, it can be postulated that the *VaDJI* overexpression augmented the activation of defense system related with antioxidants and abiotic stress, which further guarded the transgenic lines against injuries caused by ROS in drought stress in transgenic plants.

## 5 Conclusion

In the current investigation, we identified, cloned, and characterized a heat-induced *VaDJI*, which has a zinc finger motif in C-terminal domain. Its expression profile reveals that regardless of the type of stress signal, it is universally expressed during various stress treatments. Additionally, the fact that it was upregulated in response to ABA, MeJA, NaCl, PEG and ethephon treatment implies that it is a part of a network related to multiple signalling pathways. In tobacco, ectopic expression of *VaDJI* conferred ABA insensitivity. Transgenic plants demonstrated improved photosynthetic performance as well as better growth and tolerance to heat and drought stress. We suggest that *VaDJI* safeguards the photosynthetic apparatus under stress by lowering otherwise excessive ROS levels and ensures retrograde signalling from organelles like chloroplasts, as shown by the transcriptional upregulation of stress-related genes. Further research is needed to determine the upstream and downstream targets of *VaDJI* to fully clarify its biological function in ABA signalling and drought stress. Also, the results presented in the current investigation provide evidence only for a role of *VaDJI* in heat shock responses in tobacco, and that more studies will be needed to determine the similar role of the protein in *Vigna aconitifolia*.This study offers a useful resource for the possible genetic enhancement of drought and heat stress tolerance in agriculturally significant crops to withstand stress.

## Data availability statement

The original contributions presented in the study are included in the article/**Supplementary Material**. Further inquiries can be directed to the corresponding authors.

## Author contributions

PK, RG and RM designed the study. RG, RM and SR performed the experiments. The manuscript was written by PK, RG, RM and PS. All authors contributed to the article and approved the submitted version.
