# Supplementary material for: Ectopic expression of DnaJ type-I protein homolog of Vigna aconitifolia (VaDJI) confers ABA insensitivity and multiple stress tolerance in transgenic tobacco plants
Source: Front Plant Sci. 2023 Apr 19;14:1135552. doi: 10.3389/fpls.2023.1135552 (PMC10154610; doi:10.3389/fpls.2023.1135552)
Supplement: Supplementary file 1 [file DataSheet_1.pdf]

Title: Ectopic expression of DnaJ type-I protein homolog of *Vigna aconitifolia* (*VaDJI*) confers ABA insensitivity and multiple stress tolerance in transgenic tobacco plants

Ranjana Gautam[1,2*], Rajesh Kumar Meena[1], Sakshi Rampuria[1], Pawan Shukla[3] and P.B. Kirti[1*†]

Supplementary Table 1: List of primers used in the present study.

**Primer**	**Sequence**
VaDJI F<br>*VaDJI* R	TCTTTGGCGGTGGGAGT<br>CCAGACTTAGAACCCTTGCC
nptII F<br>*nptII* R	TAAAGCACGAGGAAGCGGTC<br>GATGGATTGCACGCAGGTTC
Actin F<br>Actin R	TGGCATCACACTTTCTACAA<br>CAACGGAATCTCTCAGCTCC
DREB3 F<br>*DREB3* R	ATGGCTTGGCACTTTCCCTT<br>ATATTCTTGGCGTCGGAGGA
ERF5 F<br>*ERF5* R	GGATTGTCTCCTGCTGCTGT<br>GCTCTTCTAATAACTCAGCACCC
APX F<br>*APX* R	GTTTGGGCTTTTCTCCTCGAC<br>GGAGCATAAGAGGAGCGCAA
*MnSOD*F<br>*MnSOD* R	TCCCCTACGACTATGGAGCA<br>CGGTATGCAATTTGGCGACG
CAT F<br>*CAT* R	GGCCGCTACAACTCTCTCTTT<br>ACAGGACCTCTTGCACCAAC
ERD10 F<br>*ERD10* R	GCACGAGGGAAGAAGAGAAGG<br>TGGAGGCGCCACTTCCTC
P5CS1 F<br>*P5CS1* R	GCTGCTCAACAGGCTGGATA<br>CCATCAGCAACCTCCGTTCT
SOS1 F<br>*SOS1* R	CAAATGTTATCCCCCGAAAGC<br>CGGAGAACCTGAGGAAATGTGA
Ubiquitin F<br>*Ubiquitin* R	GAGTCAACCCGTCACCTTGT<br>ACATCTTTGAGACCTCAGTAGACA

MFGRAPKKSDNTRYYEILGVSKNASQDDLKKAYKKAAIKNHPDKGGDPEKFKELAQAYEVLSDPEKREIYDQ
YGEDALKEGMGGGGGHDPFDIFSSFFGGGSPFGSGGSSRGRRQRRGEDVVHPLKVSLEDLYLGTSKKLSL
SRNVICSKCSGKGSKSGASMKCAGCQGTGMKVSIRHLGPSMIQQMQHACNECKGTGETINDRDRCPQCK
GENVVQEKKVLEVIVEKGMQNGQKITFPGEADEAPDTITGDIVFVLQQKEHPKFRRKAEDLYVEHTLSLTEAL
CGFQFVLTHLDGRQLLIKSNPGEVVKPDSYKAINDEGMPMYQRPFMKGKLYPHFTVEFPDFLNPEQVKALE
AALPPKQPSQLTDMELDECEETTLHDVNMDEESRRRQQQAAQEAYEEDDDMPGGAQRVQCAQQ

Supplementary Figure 1. Schematic representation of presence of characteristic domain of *DnaJ* type-I in VaDJI protein; DnaJ domain- represented by underline solid Black color spanning a region of 13-71 amino acids (a.a.) containing HPD motif, G/F-rich domain- represented by blue color alphabets having DIF motif, Zinc finger domain- having characteristic CSKCSGKG and CAGCQGTG, CNECKGTGE marked by red colour alphabets, C terminal domain- represented by underlined dotted black color (121-343 a.a.).

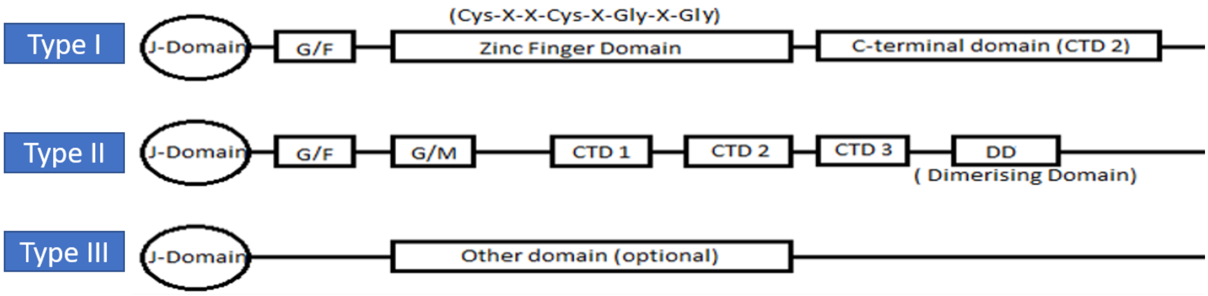

Supplementary Figure 2: Schematic diagrammatic representation of comparative domain structure of general DnaJ types.

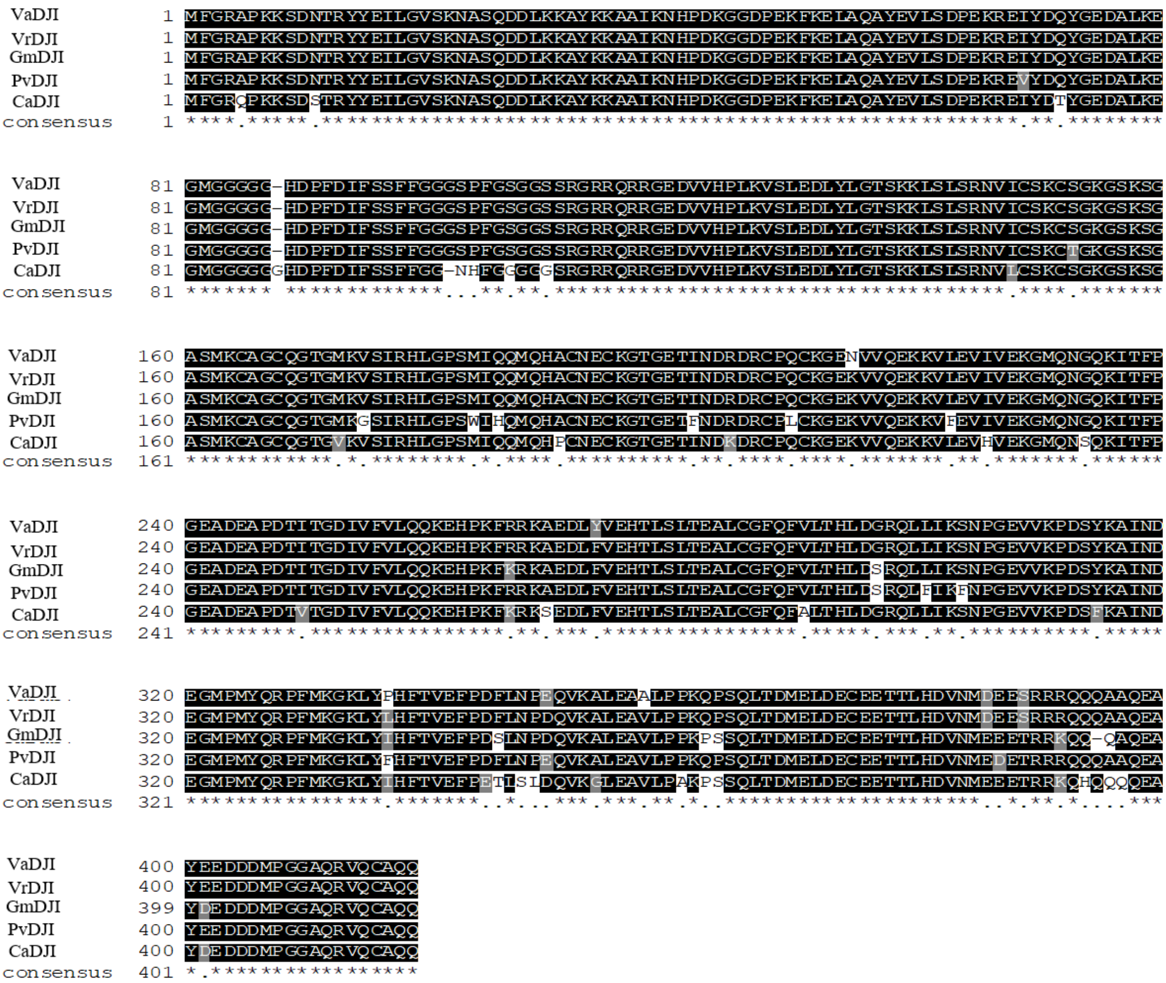

Supplementary Figure 3. Alignment of deduced amino acid sequence of VaDJI protein with other closely related DnaJ proteins from other plant species. Va- *Vigna aconitifolia*, Vr-*Vigna radiata*, Gm-*Glycine max*, Pv-*Phaseolus vulgaris*, Ca-*Cajanus albicans*.

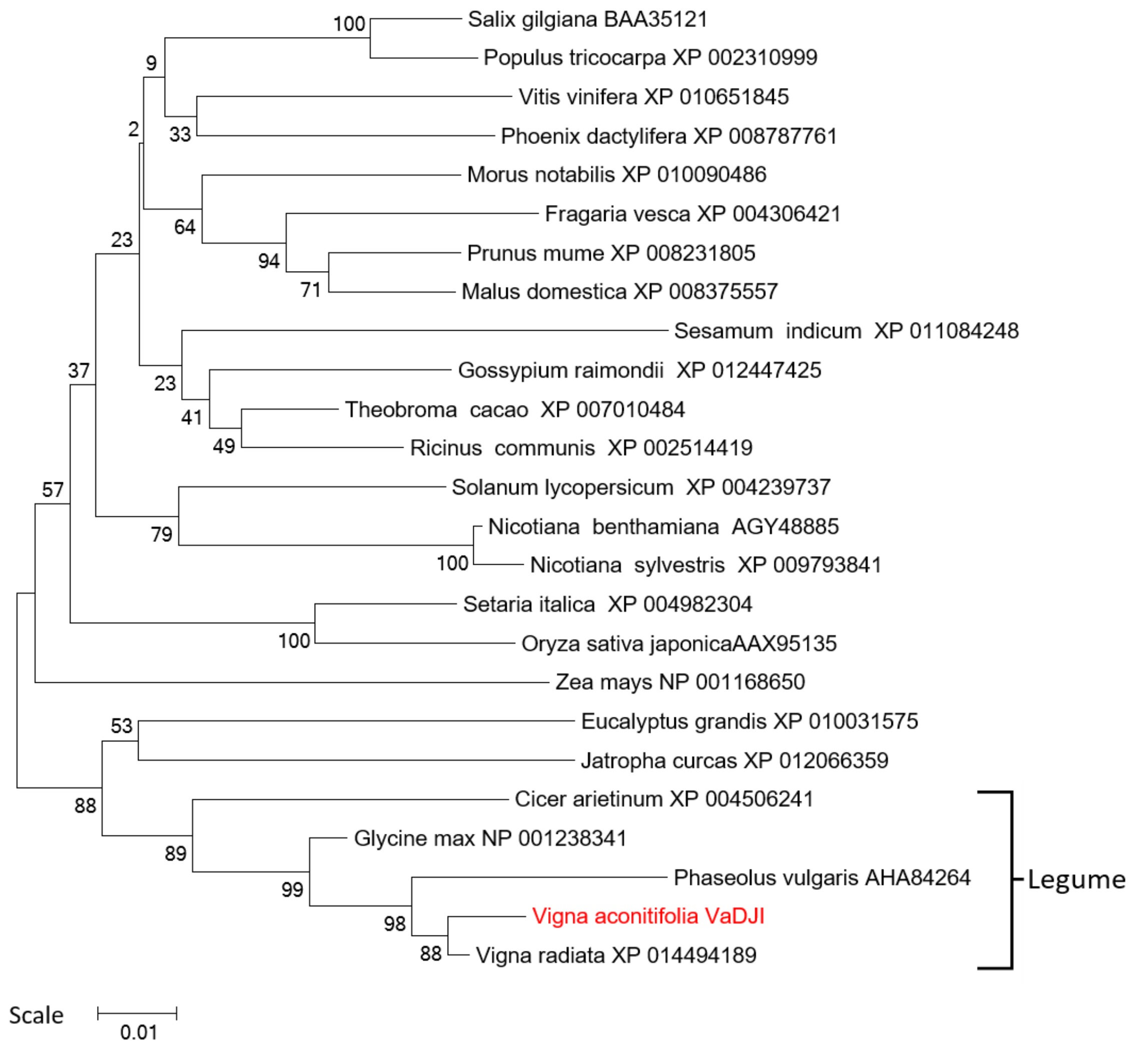

Supplementary Figure 4. Phylogenetic analysis of the DnaJ proteins. Full-length protein sequence alignment was done using the ClustalW tool, and a tree was built by using neighbor-joining (NJ) algorithm of MEGA 7.0 software which led to the classification of *VaDJI* as a type-I J protein.

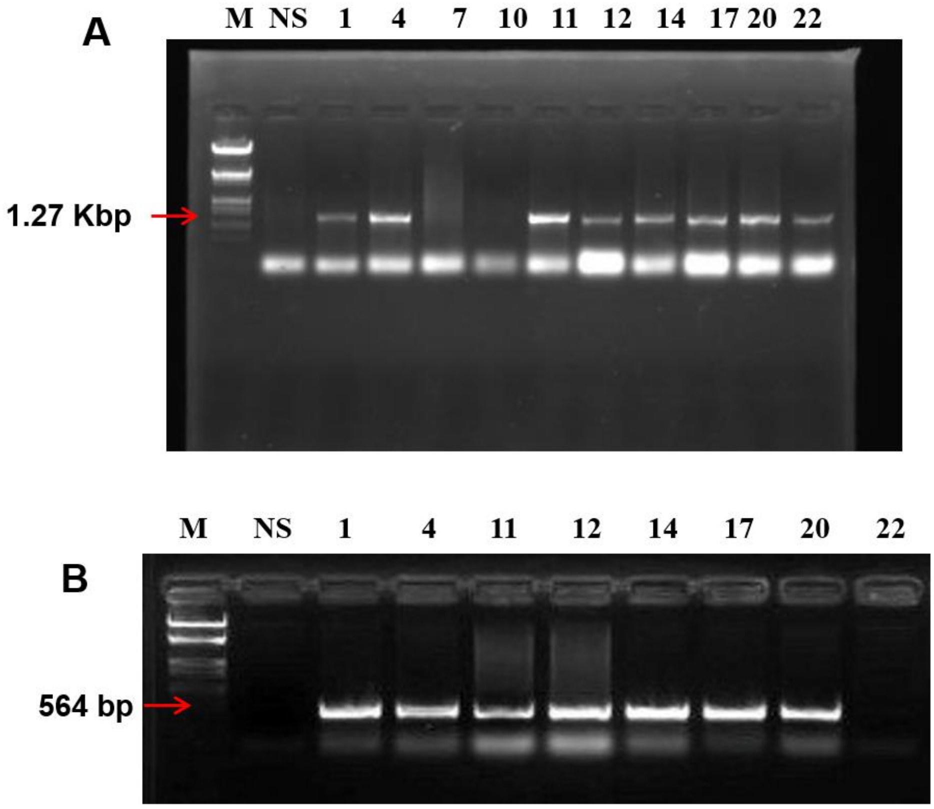

Supplementary Figure 5. Generation and molecular identification of *VaDJI* tobacco transgenics three-week-old T_2 transgenic tobacco plants via PCR; **(A)** Schematic diagram of PCR analysis from genomic DNA of T_2 transgenic tobacco using *VaDJI* gene specific primer (1.27 kb), **(B)** *nptII* gene specific primers (564 bp). Lane 1: M represents λ. *Eco*RI/*Hin*dIII DNA Marker, Lane 2: represents -ve (negative control) Lane 3-9 represents genomic DNA from T_2 transgenic tobacco plants.

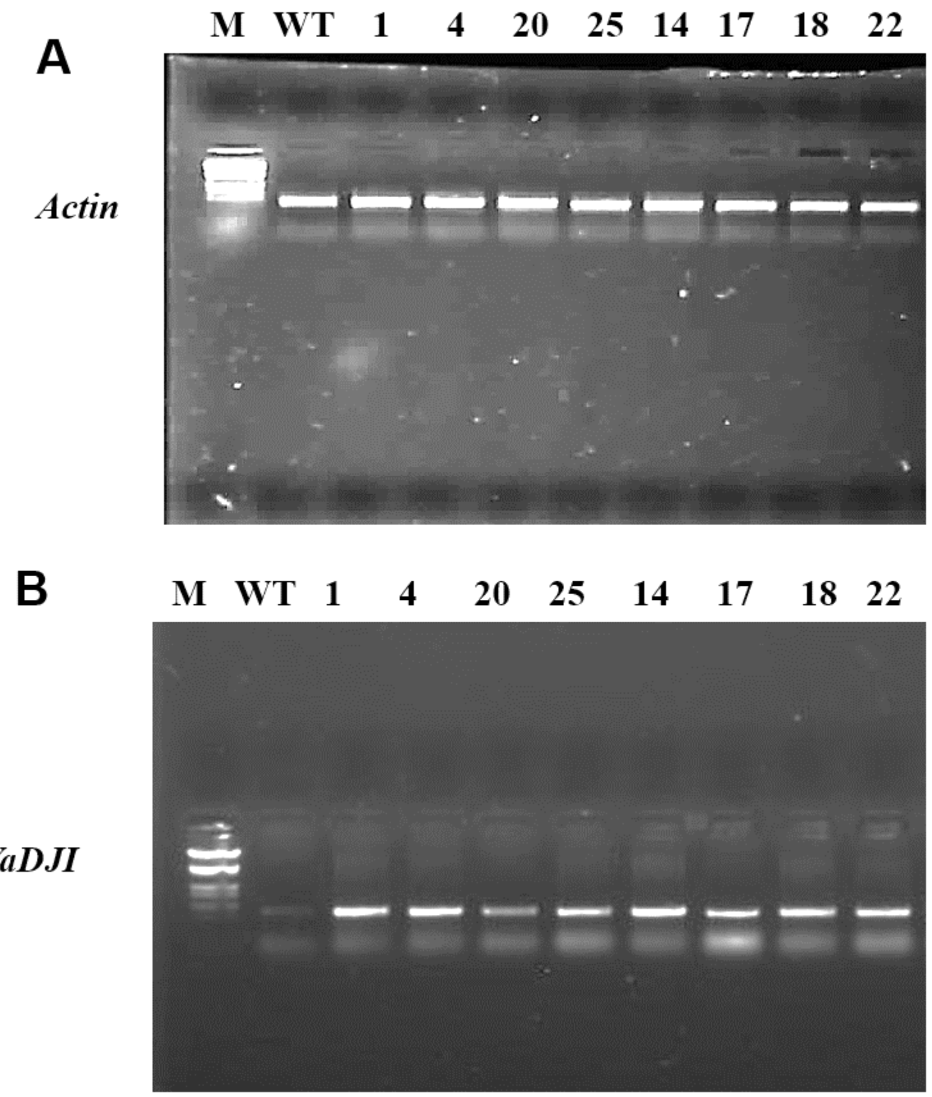

Supplementary Figure 6. Semi-Quantitative analysis for *VaDJI* expression in T_2 transgenic lines using gene specific primers for *VaDJI* and *actin* (as an internal reference). Lane 1: M represents *Eco*RI/*Hin*dIII DNA Marker, Lane2: represents cDNA from wild type plant. Lane 3-10: represents cDNA from transgenic lines.

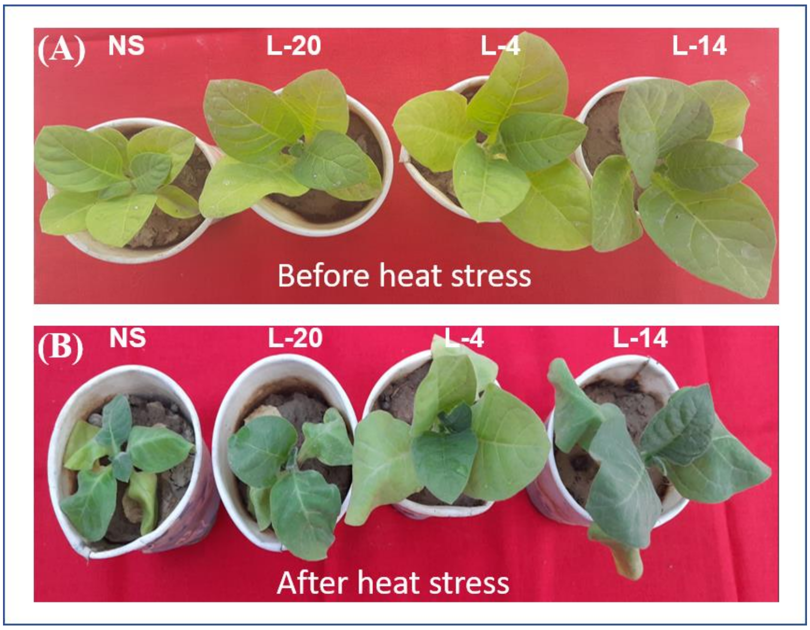

Supplementary Fig. 7. Assessment of heat tolerance observed in five-week NS and *VaDJI* transgenic tobacco plants subjected to heat stress at pot level; **(A)** Control, **(B)** Phenotypic differences after 3 h of heat stress. NS plants entirely wilted compared to *VaDJI* tobacco lines.

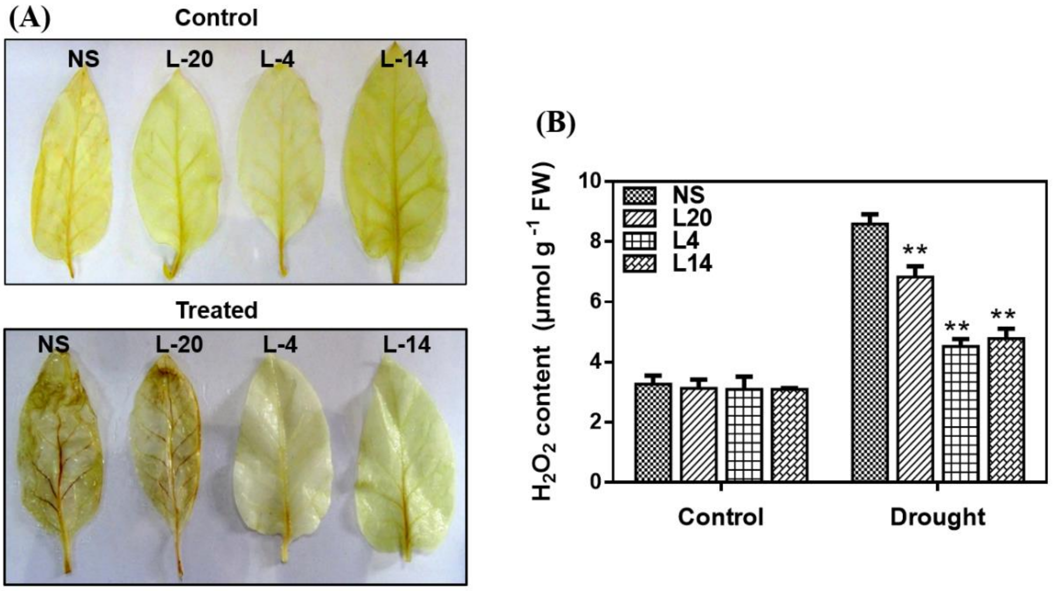

Supplementary Fig. 8. Reactive Oxygen Species (ROS) accumulation and cell damage in the NS plants and the transgenic lines; **(A)** The H_2O_2 accumulations were detected by histochemical staining in control and drought treated leaves using 3,3′-diaminobenzidine (DAB), **(B)** Quantification of H_2O_2.
